# Escore de Cálcio das Artérias Coronárias, Fatores de Risco e Desfechos Clínicos na Doença Arterial Coronariana Não Obstrutiva: Um Estudo de Seguimento de Longo Prazo

**DOI:** 10.36660/abc.20250772

**Published:** 2026-06-12

**Authors:** Protásio Lemos Da Luz, Desiderio Favarato, Alexandre A.C. Abizaid, Luiz Antônio Machado Cesar, Carlos Vicente Serrano, Antônio Carlos Palandri Chagas, Carlos E. Rochitte, Marco A. Gutierrez

**Affiliations:** 1 Instituto do Coração do Hospital das Clínicas da Faculdade de Medicina da Universidade de São Paulo São Paulo SP Brasil Instituto do Coração do Hospital das Clínicas da Faculdade de Medicina da Universidade de São Paulo, São Paulo, SP – Brasil

**Keywords:** Cálcio, Vasos Coronários, Doença da Artéria Coronariana

## Abstract

**Fundamento:**

O valor prognóstico da mensuração do escore de cálcio das artérias coronárias (CAC) na doença arterial coronária (DAC) não obstrutiva (definida como estenose < 50%) não está suficientemente descrito.

**Objetivos:**

Investigar a associação entre a mensuração do CAC, fatores de risco cardiovascular e desfechos clínicos em pacientes com DAC não obstrutiva.

**Métodos:**

Ao todo, 2.509 pacientes foram submetidos à angiotomografia coronariana (angioTC) e acompanhados por 8,9 ± 2,6 anos. Conforme o CAD-RADS™ 2.0, a carga de placa foi classificada em ausente, leve, moderada e alta/muito alta. O desfecho primário foi um composto de mortalidade por todas as causas, síndrome coronariana aguda/infarto agudo do miocárdio e acidente vascular encefálico. A significância estatística foi estabelecida em 5%.

**Resultados:**

O escore de CAC foi 0 em 45,4% dos pacientes, 1-99 em 36,6% e ≥ 100 em 18,0%. Correspondentemente, 38,3% dos pacientes não apresentavam lesões coronarianas, 38,5% apresentavam lesões leves, 14,1% lesões moderadas e 9,2% lesões altas/muito altas. Entre os pacientes com CAC = 0, a ausência de lesões coronarianas predominou (81,2%), enquanto foi rara entre aqueles com CAC ≥ 100. O desfecho primário ocorreu em 4,9% dos pacientes, predominantemente impulsionado pela mortalidade por todas as causas. Entre os 396 pacientes com angioTC coronariana seriada (intervalo médio: 6,5 ± 2,6 anos), a progressão do CAC (> 2,5 de aumento com base no método da raiz quadrada) foi observada em 41,9%. A carga de placa aumentou em paralelo. O escore de CAC apresentou associação positiva com a carga de placa e com fatores de risco cardiovascular.

**Conclusões:**

Na DAC não obstrutiva, o CAC está presente em mais da metade dos pacientes e está associado tanto à carga de placa quanto aos fatores de risco cardiovascular. A incidência do desfecho primário aumenta proporcionalmente à carga de fatores de risco e aos níveis de CAC. O CAC e a carga de placa progridem concomitantemente ao longo do tempo, sustentando o papel do CAC como marcador substituto da progressão da aterosclerose subclínica.

**Figure:**
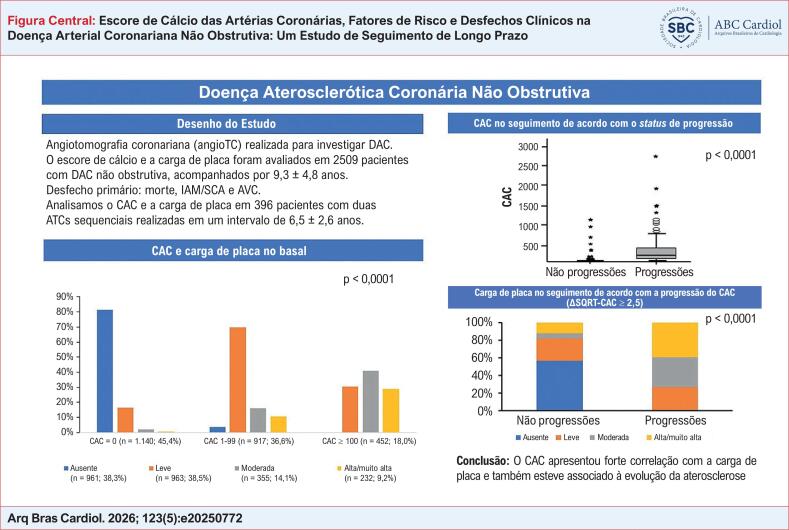


## Introdução

O cálcio das artérias coronárias (CAC) é um marcador bem estabelecido de aterosclerose e um robusto indicador prognóstico na doença arterial coronariana (DAC). Além disso, melhora a estratificação de risco quando incorporado aos modelos tradicionais de risco cardiovascular.^1–4^ A calcificação é um processo biológico ativo, amplamente impulsionado pela formação óssea ectópica, e reflete a interação entre estresse oxidativo, inflamação e remodelamento vascular.^5,6^ No entanto, a ausência de CAC não exclui aterosclerose, uma vez que muitas placas de alto risco permanecem não calcificadas.^7,8^ O CAC está associado a fatores de risco tradicionais, à carga global de placa e a eventos cardiovasculares, particularmente em valores superiores a 300 unidades de Agatston.^9–12^

Apesar de seu papel estabelecido, o CAC tem sido insuficientemente investigado em pacientes com DAC não obstrutiva, especialmente no contexto de seguimento de longo prazo. A prevalência de DAC não obstrutiva varia amplamente e é consistentemente maior em mulheres do que em homens.^13–15^ Em pacientes com DAC estável, sua prevalência foi de até 67%.^13^ Em relação aos desfechos clínicos, Wang et al.^13^ relataram taxas anuais de eventos de 0,3% em indivíduos sem DAC, 0,7% naqueles com DAC não obstrutiva e 2,7% em pacientes com DAC obstrutiva. De modo geral, o risco associado à DAC não obstrutiva é aproximadamente 72% menor do que aquele observado na DAC obstrutiva.^13^

Historicamente, pacientes com DAC não obstrutiva têm sido considerados de baixo risco. No entanto, evidências mais recentes contestam essa suposição, demonstrando que a DAC não obstrutiva está associada à mortalidade por todas as causas, infarto agudo do miocárdio (IAM) e acidente vascular encefálico (AVE).^8,16^

Dados sobre DAC não obstrutiva e CAC na população brasileira permanecem limitados ou inexistentes. Essa população é caracterizada por um background genético singular, resultante da miscigenação entre indivíduos de ancestralidade africana, europeia e indígena americana.^17,18^ Estudos prévios, particularmente aqueles conduzidos por Mayana Zatz, destacaram a importância dessa diversidade genética, que pode influenciar a expressão fenotípica. Portanto, achados derivados de outras populações podem não ser totalmente generalizáveis para indivíduos brasileiros.

Dessa forma, o presente estudo avaliou a presença e o significado clínico da mensuração do CAC, bem como sua progressão ao longo do tempo, em uma coorte de pacientes brasileiros com DAC não obstrutiva durante seguimento de longo prazo (Figura Central).

## Métodos

### População

A população do estudo consistiu em 2.509 pacientes consecutivos com idade entre 18-80 anos, incluindo homens e mulheres, submetidos à angiotomografia coronariana (angioTC) no Instituto do Coração (InCor) da Faculdade de Medicina da Universidade de São Paulo, Brasil, para avaliação de dor torácica ou suspeita de DAC, entre janeiro de 2012 e dezembro de 2017. Todos os pacientes faziam parte do registro institucional do InCor.

Um total de 803 pacientes (32,0%) foi acompanhado por meio de consultas clínicas presenciais no InCor. Os 1.706 pacientes restantes (68,0%) foram contatados por telefone ou e-mail e, após consentimento informado, responderam a um questionário padronizado (Apêndice A) sobre desfechos clínicos. Em casos de óbito, as informações foram obtidas com um familiar ou com o médico assistente do paciente. Como as causas específicas de morte frequentemente eram de difícil determinação, considerou-se apenas mortalidade por todas as causas.

Os critérios de exclusão incluíram síndrome coronariana aguda (SCA) prévia no basal, revascularização miocárdica prévia, doença valvar, cardiomiopatias, doença pulmonar obstrutiva crônica, doença renal crônica, neoplasia, insuficiência hepática ou qualquer condição associada a expectativa de vida < 5 anos.

O desfecho primário foi um composto de mortalidade por todas as causas incidente, SCA/IAM e AVE. Os desfechos secundários corresponderam aos componentes individuais do desfecho primário.

O protocolo do estudo foi aprovado pelo comitê de ética em pesquisa em seres humanos do InCor e registrado no ClinicalTrials.gov sob o código NCT05392491.

### Análise coronariana

A angioTC foi realizada utilizando um tomógrafo com 320 detectores (cobertura de 16 cm; Canon Aquilion) em 90% dos pacientes e um tomógrafo com 64 detectores nos 10% restantes, seguindo protocolos padrão de aquisição. A mensuração do CAC foi realizada previamente à angioTC por meio de aquisição axial sincronizada à eletrocardiografia, com espessura de corte de 3 mm, sem sobreposição ou intervalos.

A calcificação foi definida como uma lesão hiperatenuante > 130 unidades Hounsfield com área ≥ 1 mm^2^ ou envolvendo pelo menos três pixels contíguos. O CAC foi quantificado pelo método de Agatston.^19^

As imagens de angioTC foram interpretadas por operadores experientes utilizando técnicas padronizadas. Cada caso foi revisado independentemente por dois analistas treinados, que avaliaram os laudos gerados por dois angiografistas experientes. Em caso de discordância, as imagens foram reavaliadas por dois cardiologistas seniores para obtenção de consenso. A variabilidade interobservador foi de 2,2%. Não foram relatadas complicações relacionadas à angioTC.

A critério do médico assistente, 396 pacientes (15,7%) foram submetidos a uma segunda angioTC após um intervalo médio de 6,5 ± 2,6 anos. As indicações para repetição do exame incluíram seguimento de rotina, teste funcional de isquemia alterado, sintomas cardíacos ou avaliação pré-operatória para cirurgia não cardíaca (71,6%); em 28,8% dos casos, a indicação não estava disponível.

A progressão do CAC foi avaliada pelo método da raiz quadrada (SQRT, do inglês *square root*) e definida como um aumento > 2,5 no escore de CAC transformado por SQRT entre a segunda e a primeira angioTC. Esse método foi demonstrado por Budoff et al.^20^ como uma medida sensível de progressão do CAC. A carga de placa, os fatores de risco e os desfechos clínicos foram posteriormente comparados entre progressões e não progressões.

### Classificação dos territórios coronarianos

Para análise, a circulação coronariana foi dividida nos seguintes territórios: i) tronco da coronária esquerda e artéria descendente anterior, incluindo ramos diagonais e septais; ii) artéria circunflexa e ramos marginais (Mg1 e Mg2); e iii) artéria coronária direita e seus ramos.

### Classificação da doença arterial coronariana e carga de placa

Os pacientes foram categorizados nos seguintes grupos: i) ausência de DAC aparente e ii) presença de DAC, classificada de acordo com um sistema CAD-RADS™ 2.0 modificado.^21^

A gravidade das lesões foi definida da seguinte forma: i) ausente (0% de estenose); ii) leve (≤ 30% de estenose); e iii) moderada (> 30%-49% de estenose).

A carga de placa foi classificada como: i) ausente: sem lesões; ii) leve: até duas lesões leves (≤ 30%); iii) moderada: três lesões leves (≤ 30%) ou até duas lesões moderadas (> 30%-49%); iv) alta: acometimento do tronco da coronária esquerda ou pelo menos três lesões moderadas (> 30%); v) muito alta: progressão além da categoria "alta" ou presença de pelo menos uma lesão ≥ 50% na segunda angioTC.

As lesões coronarianas foram avaliadas visualmente com base no diâmetro luminal do segmento doente em relação ao segmento de referência proximal mais normal.

### Dados demográficos

Os seguintes dados basais foram coletados na inclusão no estudo (Tabela 1 e Apêndice B): idade, sexo, peso, pressão arterial e altura. O índice de massa corporal (IMC) foi calculado a partir do peso e da altura.

**Tabela 1:** Características demográficas e clínicas basais da população do estudo (n = 2.509)

Característica		n (%)	Dados ausentes
**Sexo**		
	Feminino	1.167 (46,5%)	—
	Masculino	1.342 (53,5%)	—
**Raça**			337
	Branco	1.924 (88,6%)	—
	Negro	81 (3,7%)	—
	Pardo	90 (4,1%)	—
	Asiático	77 (3,5%)	—
**Idade, mediana (IIQ), anos**		58 (50,43-64,60)	1
**Histórico familiar positivo de DAC**		1.201 (56,1%)	367
**DM**		896 (35,7%)	—
**Dislipidemia**		1.817 (72,4%)	—
**HAS**		1.788 (71,3%)	—
**Estilo de vida sedentário**		1.105 (47,3%)	171
**Obesidade**		638 (27,0%)	146
**Tabagismo**			101
	Ex-tabagista	772 (32,1%)	—
	Tabagista atual	199 (8,6%)	—
**Classificação da carga de placa**			—
	Ausente	961 (38,3%)	—
	Leve	963 (38,4%)	—
	Moderada	353 (14,1%)	—
	Alta/muito alta	232 (9,2%)	—
**Medicações**			—
	Anti-hipertensivos	1.722 (68,6%)	—
	Antidiabéticos	837 (33,4%)	—
	Terapia hipolipemiante	1.706 (68,1%)	—

Fonte: Elaboração dos autores (2025). DAC: doença arterial coronariana; DM: diabetes melito; HAS: hipertensão arterial sistêmica; IIQ: intervalo interquartil.

Hipertensão arterial sistêmica (HAS) foi definida como pressão arterial sistólica > 130/85 mmHg ou uso de medicação anti-hipertensiva. Obesidade foi definida como IMC ≥ 30 kg/m^2^ e sobrepeso como IMC entre 26 e 29 kg/m^2^. A atividade física foi categorizada como sedentária ou ativa (≥ três sessões por semana). Histórico familiar positivo foi definido como pelo menos um dos pais com eventos cardiovasculares ou intervenções antes dos 60 anos de idade.

O tabagismo foi classificado como nunca, ex-tabagista ou atual (≥ 10 cigarros/dia). Diabetes melito (OK?) (DM) foi definido como glicemia de jejum ≥ 126 mg/dl, glicemia casual > 140 mg/dl, teste oral de tolerância à glicose após 2 horas > 200 mg/dl, HbA1c > 6,5% ou uso de medicamentos hipoglicemiantes. Dislipidemia foi definida como colesterol de lipoproteína de baixa densidade > 130 mg/dl, triglicerídeos > 150 mg/dl ou uso de terapia hipolipemiante.

### Análises estatísticas

As variáveis categóricas são apresentadas como frequências absolutas e relativas, enquanto as variáveis contínuas são expressas como média ± desvio padrão para dados com distribuição normal ou mediana e intervalo interquartil para dados com distribuição não normal, conforme avaliado pelo teste de Kolmogorov-Smirnov.

As características basais e os eventos clínicos foram inicialmente comparados sem ajuste. A incidência do desfecho primário foi avaliada por meio de curvas de sobrevida de Kaplan-Meier e comparada pelo teste *log-rank*. O tempo desde o exame inicial até a ocorrência do evento primário foi utilizado na análise de sobrevida.

As associações entre variáveis categóricas foram avaliadas pelo teste do qui-quadrado, e as diferenças na carga de placa foram analisadas pelo teste de Mann-Whitney. As mudanças no CAC entre a primeira e a segunda mensuração, expressas como valores transformados por SQRT, foram analisadas pelo teste de Wilcoxon pareado, uma vez que os valores de SQRT não apresentaram distribuição normal.

As comparações entre a angioTC basal e de seguimento em não progressões e progressões foram realizadas pelo teste de postos sinalizados de Wilcoxon. A regressão logística binária foi utilizada para identificar fatores associados à progressão do CAC, com resultados expressos como *odds ratio* (OR) e intervalo de confiança de 95% (IC95%). O modelo final foi selecionado por abordagem *stepwise*, incluindo variáveis com p < 0,20 na análise univariada (dados não apresentados) ou variáveis já estabelecidas como fatores de risco cardiovascular tradicionais.

A colinearidade foi avaliada por meio do fator de inflação da variância, sendo valores > 4 considerados indicativos de colinearidade. A associação entre o escore de CAC e a carga de placa no contexto da progressão do CAC foi avaliada pelo teste de correlação de Spearman.

## Resultados

### Características basais

Entre os 2.509 pacientes, 53,5% eram homens e 46,5% eram mulheres, com idade média de 57,2 ± 10,9 anos. A maioria dos pacientes era branca e apresentava múltiplos fatores de risco cardiovascular, incluindo HAS, dislipidemia, sobrepeso/obesidade e histórico familiar positivo (Tabela 1).

**Figura 1:**
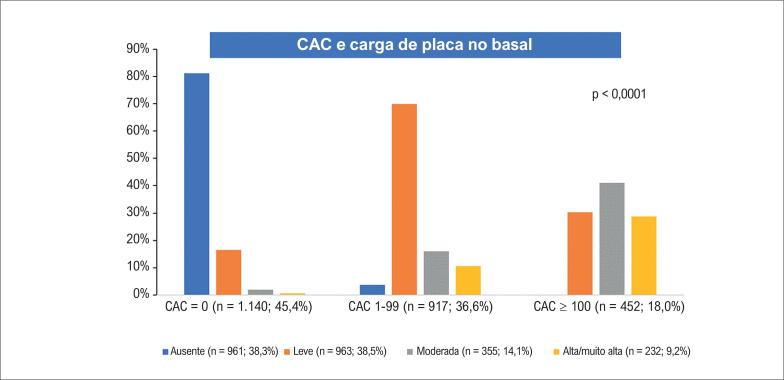
Comparação entre as categorias de CAC e a carga de placa (ausente, leve, moderada, alta/muito alta) na população total (n = 2.509). Observa-se um gradiente claro na carga de placa com o aumento das categorias de CAC, de 0 a ≥ 100. Entre os pacientes com CAC = 0, predomina a ausência de lesões coronarianas, enquanto lesões leves, moderadas e altas/muito altas predominam entre aqueles com CAC ≥ 100 unidades de Agatston (teste do qui-quadrado de Pearson). Ver texto para detalhes. CAC: cálcio das artérias coronárias.

No basal, o CAC foi 0 em 1.140 pacientes (45,4%), 1-99 em 917 (36,6%) e ≥ 100 em 452 (18,0%); neste último grupo, o valor médio de CAC foi 368,4 ± 347,3. Concomitantemente, 961 pacientes (38,3%) não apresentavam lesões coronarianas, 963 (38,5%) apresentavam lesões leves, 355 (14,1%) lesões moderadas e 232 (9,2%) lesões altas/muito altas (Figura 1).

A carga de placa no basal foi predominantemente classificada como ausente ou leve, enquanto apenas 9,2% dos pacientes apresentaram carga de placa alta/muito alta (Tabela 1).

### Cálcio das artérias coronárias e carga de placa

Houve uma relação direta clara entre CAC e carga de placa no basal (Figura 1). Entre os pacientes com CAC = 0, a ausência de lesões coronarianas predominou (80%), enquanto nenhum paciente com CAC ≥ 100 apresentou lesões nulas. Em contrapartida, as proporções de lesões leves, moderadas e altas/muito altas aumentaram progressivamente com valores mais elevados de CAC, atingindo 100% entre os pacientes com CAC ≥ 100.

### Subgrupos de cálcio das artérias coronárias e fatores de risco

O número de fatores de risco cardiovascular aumentou ao longo das categorias de CAC. Sexo masculino, idade > 65 anos, DM, dislipidemia e HAS foram significativamente mais frequentes entre os pacientes com CAC ≥ 100 em comparação àqueles com CAC = 0 (p < 0,001, teste do qui-quadrado).

Embora o número de fatores de risco tenha aumentado com o aumento do CAC, mesmo entre pacientes com CAC = 0, 69,3% apresentavam três ou > três fatores de risco (Tabela 2).

**Tabela 2:** CAC, fatores de risco cardiovascular e taxas de eventos no basal

Variável		CAC = 0 (n = 1.140)	0 < CAC < 100 (n = 917)	CAC ≥ 100 (n = 452)	Total (n)	Valor de p^1^
Fatores de risco						
**DM**		341 (29,9%)	365 (39,8%)	190 (42,0%)	2.509	< 0,001
**Dislipidemia**		725 (63,6%)	721 (78,6%)	371 (82,1%)	2.509	< 0,001
**HAS**		732 (64,2%)	689 (75,1%)	367 (81,2%)	2.509	< 0,001
**Histórico familiar de DAC**		542 (54,5%)	452 (58,6%)	207 (54,9%)	2.142	0,201
**Tabagismo atual**		93 (8,4%)	68 (7,8%)	38 (8,7%)	2.408	0,809
**Estilo de vida sedentário**		496 (47,3%)	411 (47,7%)	198 (46,4%)	2.338	0,906
**Idade > 65 anos**		140 (12,3%)	259 (28,2%)	199 (44,0%)	2.508	< 0,001
**Sexo masculino**		539 (47,3%)	494 (53,9%)	309 (68,4%)	2.509	< 0,001
**Carga de fatores dez risco**						< 0,0001
	0	17 (1,5%)	1 (0,1%)	0 (0,0%)	—	—
	1	101 (8,9%)	30 (3,3%)	8 (1,8%)	—	—
	2	232 (20,3%)	118 (12,9%)	32 (7,0%)	—	—
	3	327 (28,7%)	223 (24,3%)	97 (21,5%)	—	—
	> 3	463 (40,6%)	545 (59,4%)	311 (69,7%)	—	—
**Taxas de eventos por categoria de CAC**						
	Desfecho primário	42 (3,7%)	44 (4,8%)	37 (8,2%)	—	< 0,0001
	SCA/IAM	15 (1,3%)	14 (1,5%)	10 (2,2%)	—	0,365
	AVE	17 (1,5%)	19 (2,1%)	13 (2,9%)	—	0,141
	Mortalidade por todas as causas	14 (1,2%)	12 (1,3%)	19 (4,2%)	—	< 0,0001

Os participantes foram estratificados de acordo com as categorias de CAC (0, 0 < CAC < 100 e CAC ≥ 100). Os valores são expressos como números absolutos e porcentagens. As comparações entre grupos foram realizadas pelo teste do qui-quadrado^1^. A sobrevida livre de eventos foi analisada por curvas de Kaplan-Meier com o teste *log-rank*. AVE: acidente vascular encefálico; CAC: cálcio das artérias coronárias; DAC: doença arterial coronariana; DM: diabetes melito; HAS: hipertensão arterial sistêmica; IAM: infarto agudo do miocárdio; SCA: síndrome coronariana aguda.

### Fatores associados à progressão do cálcio das artérias coronárias

Entre 396 pacientes (15,8% da coorte total) que foram submetidos a uma segunda angioTC após um intervalo médio de 6,5 ± 2,6 anos, 165 (41,7%) foram classificados como progressões de acordo com o método SQRT, enquanto 231 (58,3%) foram classificados como não progressões.

A mediana (intervalo interquartil) do escore de CAC aumentou de 0 (0-31) no basal para 12,6 (5,0-126,75) no seguimento (p < 0,0001, teste de Wilcoxon pareado).

Em relação às transições entre categorias de CAC, 71,9% dos pacientes com CAC = 0 permaneceram inalterados, 26,7% progrediram para CAC 1-99 e 1,4% progrediram para CAC ≥ 100. Entre os pacientes com CAC de 1-99, 31,0% progrediram para CAC ≥ 100.

As características basais de progressões e não progressões são apresentadas na Tabela 3. Na análise univariada, sexo masculino, idade (tanto como variável dicotômica > 65 anos quanto como variável contínua), HAS, DM, dislipidemia e uso de terapias anti-hipertensiva, antidiabética e com estatinas estiveram associados à progressão do CAC (Tabela 3). Na análise multivariada, apenas sexo masculino e idade permaneceram independentemente associados à progressão (Apêndice B).

**Tabela 3:** Características demográficas e clínicas de não progressões e progressões

Característica		Não progressões (n = 231)	Progressões (n = 165)	OR (IC95%)	Valor de p	Dados ausentes
**Número (%)**		231 (58,3%)	165 (41,7%)	—	—	—
**Tempo até a segunda angioTC, anos (média ± DP)**		6,49 ± 2,55	6,87 ± 2,66	—	0,264	0
**Sexo (masculino/feminino), n (%)**		104/127 (45,0/55,0)	102/63 (61,8/38,2)	1,98 (1,32-2,97)	0,0009	0
	Idade ≥ 65 anos, n (%)	32 (13,9%)	38 (23,0%)	1,86 (1,11-3,13)	0,018	0
	Idade, anos (média ± DP)	52,38 ± 3,68	57,79 ± 9,38	—	< 0,0001	0
**Histórico familiar de DAC, n (%)**		116 (57,1%)	80 (59,7%)	1,11 (0,71-1,73)	0,640	59
	HAS, n (%)	80 (34,6%)	125 (75,8%)	5,89 (3,77-9,23)	< 0,0001	0
	DM, n (%)	59 (25,5%)	77 (46,7%)	2,55 (1,67-3,90)	< 0,0001	0
	Dislipidemia, n (%)	175 (75,8%)	144 (87,3%)	2,19 (1,27-3,80)	0,004	0
***Status* tabagista, n (%)**						**37**
	Atual	14 (6,5%)	16 (11,1%)	1,76 (0,83-3,72)	0,136	—
	Ex-tabagista	52 (24,2%)	43 (29,9%)	1,29 (0,80-2,08)	0,287	—
	Nunca	150 (70,3%)	85 (59,0%)	0,68 (0,43-1,06)	0,085	37
**Atividade física, n (%)**		**91 (50,0%)**	**79 (56,0%)**	**1,27 (0,82-1,98)**	**0,280**	**73**
**Medicações, n (%)**						
	Anti-hipertensivos	148 (64,1%)	121 (73,3%)	1,54 (0,99-2,39)	0,052	0
	Antidiabéticos	48 (20,7%)	70 (42,4%)	2,81 (1,80-4,38)	< 0,0001	0
	Terapia com estatinas	148 (64,1%)	139 (84,2%)	2,46 (1,48-4,08)	0,0004	0

Características demográficas e clínicas comparando não progressões (n = 231) e progressões (n = 165). Algumas variáveis apresentaram dados ausentes, incluindo histórico familiar, *status* tabagista e atividade física. O tempo até a segunda angioTC apresentou distribuição normal (teste de Kolmogorov-Smirnov). Fonte: Elaboração dos autores (2025). DAC: doença arterial coronariana; DM: diabetes melito; DP: desvio padrão; HAS: hipertensão arterial sistêmica; IC95%: intervalo de confiança de 95%; OR: *odds ratio*.

**Figura 2:**
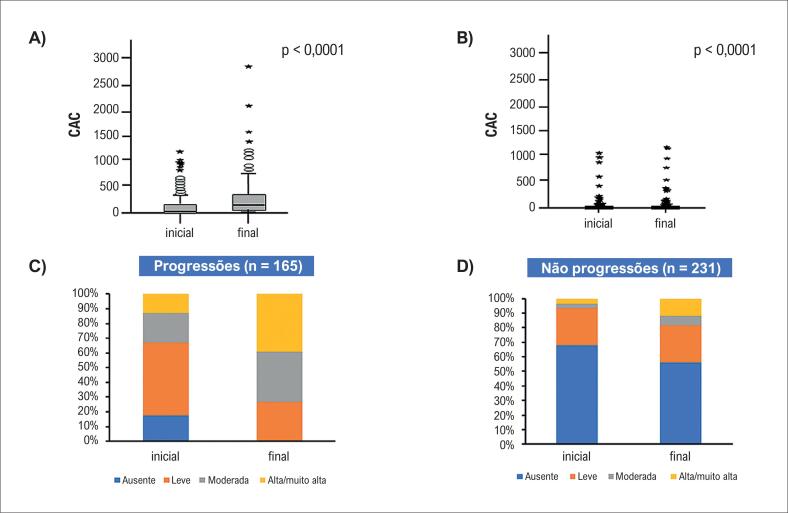
A) CAC basal e no seguimento em progressões; B) CAC basal e no seguimento em não progressões; C) carga de placa basal e no seguimento em progressões (n = 165); D) carga de placa basal e no seguimento em não progressões (n = 231). A progressão do CAC foi definida como um aumento ≥ 2,5 no CAC transformado por SQRT entre a primeira e a segunda angioTC. Ambos os grupos apresentaram mudanças significativas na carga de placa no seguimento em comparação ao basal, embora a magnitude da mudança tenha sido menor nas não progressões (teste de McNemar). AngioTC: angiotomografia coronariana; CAC: cálcio das artérias coronárias; SQRT: raiz quadrada.

O CAC aumentou significativamente da primeira para a segunda angioTC entre as progressões (de 120,4 ± 206,3 para 283,4 ± 396,7 unidades de Agatston; p < 0,001). Um aumento menor, ainda que estatisticamente significativo, também foi observado entre as não progressões (de 27,0 ± 120,0 para 32,3 ± 131,6 unidades de Agatston; p < 0,0001) (Figura 2A-B).

Utilizando a transformação SQRT, as progressões apresentaram aumento de 5,1 (0,0-12,47) para 11,45 (6,02-18,99) (p < 0,0001), excedendo o ponto de corte predefinido de 2,5. Em contraste, as não progressões apresentaram aumento menor, de 0,0 (0,0-0,0) para 0,0 (0,0-2,16) (p < 0,001), permanecendo abaixo do ponto de corte (Figura 2A-B).

**Figura 3:**
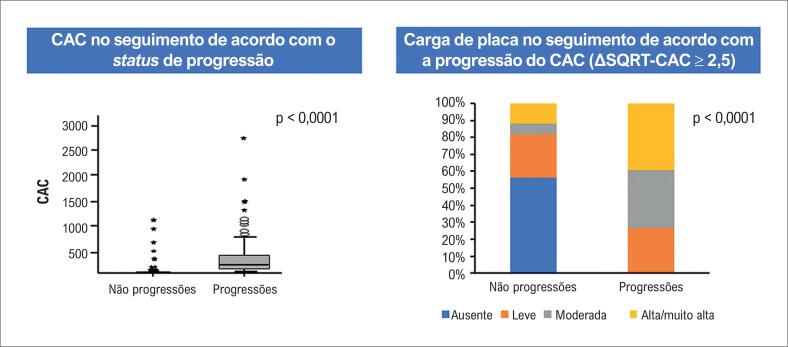
Comparação do CAC no seguimento e da carga de placa entre progressões e não progressões. As progressões apresentaram valores significativamente mais elevados de CAC e maior carga de placa em comparação às não progressões (teste de Mann-Whitney; p < 0,001 para ambos). Ver texto para detalhes. CAC: cálcio das artérias coronárias; SQRT: raiz quadrada.

Os valores finais de CAC e de carga de placa foram significativamente maiores nas progressões em comparação às não progressões (p < 0,001 para ambos; testes do qui-quadrado e Mann-Whitney) (Figura 3). De modo geral, tanto o CAC quanto a carga de placa aumentaram ao longo do tempo, com maiores incrementos observados entre as progressões.

Observou-se correlação positiva entre as mudanças no CAC e na carga de placa tanto nas progressões quanto nas não progressões (r = 0,571 e r = 0,558, respectivamente; p < 0,001 para ambos, teste de Spearman).

### Cálcio das artérias coronárias e desfecho primário

No total, 123 pacientes (4,9%) apresentaram o desfecho primário ao longo de um seguimento médio de 8,9 ± 2,6 anos. A distribuição dos eventos (mortalidade por todas as causas, SCA/IAM e AVE) é apresentada na Tabela 2.

Na análise multivariada incluindo categorias de CAC e todos os fatores de risco, histórico familiar, idade > 65 anos e estilo de vida sedentário estiveram independentemente associados ao desfecho primário (Tabela 4). Quando as categorias de CAC e o número de fatores de risco foram analisados separadamente, ambos estiveram significativamente associados aos eventos (CAC: OR, 1,36, IC95%, 1,2-1,6; p = 0,012; fatores de risco: OR, 1,56, IC95%, 1,3-1,8; p = 0,0007) (Tabela 4).

**Tabela 4:** Análise de regressão logística multivariada para o desfecho primário

Variável	OR	IC95%	Valor de p
Model 1			
Categorias de CAC	1,11	0,80-1,53	0,512
Sexo masculino	1,66	1,21-2,27	0,027
Histórico familiar de DAC	2,20	1,73-3,00	0,001
DM	1,17	0,73-2,00	0,481
HAS	1,70	1,13-2,57	0,070
Dislipidemia	0,73	0,24-1,20	0,212
Tabagismo atual	1,70	1,00-2,90	0,140
Estilo de vida sedentário	2,33	1,89-3,00	< 0,0001
Idade ≥ 65 anos	4,72	4,24-5,26	< 0,0001

OR representa o aumento do risco por incremento de categoria. As categorias de CAC foram definidas como 0, 1-99 e ≥ 100. A carga de fatores de risco foi categorizada como 0, 1, 2, 3 e > 3 fatores. Fonte: Elaboração dos autores (2025). CAC: cálcio das artérias coronárias; DAC: doença arterial coronariana; DM: diabetes melito; HAS: hipertensão arterial sistêmica; IC95%: intervalo de confiança de 95%; OR: *odds ratio*.

Entre os 165 pacientes com progressão, ocorreram quatro eventos (2,4%), em comparação a seis eventos (2,6%) entre os 231 sem progressão, sem diferença significativa entre os grupos.

As taxas de eventos foram semelhantes entre homens (7,2%) e mulheres (6,1%) (p = 0,26). No entanto, pacientes com idade > 65 anos (n = 598) apresentaram maior incidência de eventos em comparação aos indivíduos mais jovens (12,4% vs 4,9%; p < 0,001).

## Discussão

No presente estudo, o CAC foi um achado frequente entre pacientes com DAC não obstrutiva, com aproximadamente metade da coorte apresentando CAC > 0 e 18,0% exibindo CAC ≥ 100. O escore de CAC esteve diretamente associado à carga de placa, aos fatores de risco cardiovascular tradicionais e à progressão da doença. Notavelmente, este estudo foi conduzido em uma população brasileira caracterizada por um *background* genético singular, resultante da miscigenação entre indivíduos de ancestralidade africana, europeia e indígena.^17,18^ Essa característica confere originalidade aos achados, uma vez que estudos de longo prazo comparáveis em populações semelhantes permanecem escassos. Estudos prévios de grande escala com seguimento prolongado demonstraram que valores elevados de CAC estão associados a eventos cardiovasculares maiores, incluindo mortalidade por todas as causas.^7,8^

Por outro lado, investigações anteriores destacaram o valor prognóstico do CAC = 0, tanto como preditor negativo de eventos cardiovasculares^22,23^ quanto como modificador de decisões terapêuticas, incluindo o uso de estatinas e semaglutida.^24,25^ No entanto, nossos achados diferem parcialmente, pois a incidência do desfecho primário entre pacientes com CAC = 0 não foi desprezível (3,7%). Essa observação provavelmente é explicada pela alta prevalência de fatores de risco nesse subgrupo, particularmente quando ≥ três fatores de risco estavam presentes, bem como pelo período de seguimento relativamente longo (8,9 anos). Esses achados reforçam que o escore de CAC não deve ser interpretado de forma isolada, mas sim em conjunto com o perfil global de fatores de risco. É importante destacar que a ausência de CAC não exclui a presença de placas ateroscleróticas, incluindo lesões potencialmente de alto risco.

A relação consistente e robusta observada entre CAC e carga de placa neste estudo confirma achados prévios.^1–3^ Enquanto CAC = 0 esteve predominantemente associado à ausência ou à carga leve de placa, CAC ≥ 100 esteve fortemente associado à carga moderada a alta de placa. Rumberger et al.^11^ demonstraram uma relação direta e estatisticamente significativa entre o escore de CAC e a carga de placa utilizando a transformação SQRT para lidar com a distribuição assimétrica dos dados. Além disso, o CAC esteve associado a maior idade, DM, HAS e dislipidemia, em concordância com estudos prévios.^10,12^

A progressão do CAC foi observada em 41,7% dos pacientes submetidos a uma segunda angioTC e esteve associada a idade avançada, dislipidemia, DM e HAS, mas não a outros fatores de risco. A progressão foi definida utilizando o método SQRT, considerado um indicador sensível da progressão da aterosclerose.^19^

De forma semelhante, Fuster et al.,^26^ em uma coorte de 732 indivíduos assintomáticos acompanhados por 12,4 anos, demonstraram que tanto a carga de placa carotídea quanto o CAC estiveram associados à mortalidade por todas as causas e à progressão da aterosclerose. Esses achados são consistentes com os de Eghtedari et al.,^27^ que estudaram 3.260 pacientes com intervalo médio de 4,7 ± 3,2 anos entre exames e seguimento de 9 anos, demonstrando que um aumento anual de 20 unidades de Agatston foi preditor de mortalidade por todas as causas.

A possível influência de terapias farmacológicas, incluindo agentes anti-hipertensivos, antidiabéticos e hipolipemiantes, não pode ser excluída. Na análise univariada, tais associações foram observadas. Especificamente, van Rosendael et al.^28^ relataram que a terapia com estatinas pode atenuar a progressão da placa. No entanto, na análise multivariada, apenas sexo masculino e idade permaneceram independentemente associados à progressão do CAC. Diante dessas incertezas, provavelmente relacionadas ao tamanho amostral limitado de pacientes com imagem seriada, não é possível estabelecer conclusões definitivas.

Notavelmente, as mudanças no escore de CAC apresentaram correlação positiva com as mudanças na carga de placa tanto em progressões quanto em não progressões, com associação mais forte entre as progressões (Tabela 3). Embora a progressão do CAC possa atuar como marcador substituto da evolução da placa, isso não se refletiu nos desfechos clínicos, uma vez que taxas semelhantes do desfecho primário foram observadas em ambos os grupos. Esses achados sugerem que o aumento do CAC reflete progressão subclínica da aterosclerose. Além disso, a associação entre progressão do CAC e fatores de risco reforça a importância do controle rigoroso desses fatores para prevenir a progressão da doença.

De modo geral, nossos achados estão em concordância com estudos prévios que demonstram que a DAC não obstrutiva está associada a desfechos cardiovasculares adversos, incluindo morte e IAM.^14,15^ O CAC esteve significativamente associado a eventos clínicos, com maiores taxas de eventos observadas entre pacientes com CAC ≥ 100 em comparação àqueles com CAC = 0. Especificamente, 3,7% dos pacientes com CAC = 0 apresentaram eventos, em comparação com 8,2% entre aqueles com CAC > 0. Esses achados são consistentes com o estudo MESA^3^ e estão alinhados com observações em pacientes com DAC obstrutiva.

A progressão paralela do escore de CAC e da carga de placa sugere que a repetição rotineira da angioTC logo após o exame inicial pode não ser necessária, exceto em cenários clínicos selecionados.

### Pontos fortes e limitações do estudo

Este estudo inclui uma grande coorte (n = 2.509) com seguimento de longo prazo (média de 8,9 anos), abrangendo homens e mulheres em ampla faixa etária e sem eventos cardiovasculares prévios. A associação observada entre o escore de CAC e desfechos clínicos reforça seu papel como marcador de aterosclerose e destaca sua utilidade potencial na orientação de estratégias preventivas, particularmente em indivíduos que, de outra forma, poderiam ser considerados de baixo risco com base apenas na avaliação clínica. Além disso, este estudo fornece dados inéditos sobre o CAC como marcador de progressão da aterosclerose em pacientes com DAC não obstrutiva em uma população brasileira.

Entretanto, algumas limitações devem ser reconhecidas. A coleta de dados baseou-se parcialmente em informações autorreferidas, o que pode introduzir viés de relato. O estudo foi conduzido em um único centro, limitando a generalização para outras populações. Ademais, o número de pacientes submetidos à angioTC repetida foi relativamente pequeno, impedindo conclusões definitivas sobre a relação entre progressão da doença e eventos clínicos. Permanece incerto se os eventos clínicos foram impulsionados pela progressão da placa ou por instabilidade da placa relacionada à disfunção endotelial microvascular e/ou trombose.

Por fim, este foi um estudo pragmático, não randomizado, no qual a indicação para angioTC foi baseada na decisão do médico assistente. Apenas pacientes com DAC não obstrutiva foram incluídos e acompanhados ao longo de um período prolongado. Nesse contexto, os achados do presente estudo podem contribuir para estratégias de manejo mais individualizadas em pacientes com DAC subclínica.

## Conclusões

O CAC é um marcador de aterosclerose coronariana e de suas complicações clínicas, além de um robusto indicador prognóstico. Está intimamente associado à carga de placa e aos fatores de risco cardiovascular tradicionais.

Além disso, o escore de CAC e a carga de placa apresentam comportamento paralelo tanto na progressão quanto na estabilização da DAC. É importante destacar que o escore de CAC não deve ser interpretado de forma isolada, mas sim em conjunto com o perfil global de fatores de risco.

Esses achados fornecem evidências adicionais sobre o papel da mensuração do CAC na DAC não obstrutiva e podem ter implicações para a tomada de decisão clínica.

## Data Availability

Todo o conjunto de dados que dá suporte aos resultados deste estudo está disponível mediante solicitação ao autor correspondente

## *Material suplementar

*Material suplementar Para o Apêndice A, por favor, clique aqui. Para o Apêndice B, por favor, clique aqui.
