# Supplementary material for: Escore de Cálcio das Artérias Coronárias, Fatores de Risco e Desfechos Clínicos na Doença Arterial Coronariana Não Obstrutiva: Um Estudo de Seguimento de Longo Prazo
Source: Arq Bras Cardiol. 2026 Jun 12;123(5):e20250772. [Article in Portuguese] doi: 10.36660/abc.20250772 (PMC13399544; doi:10.36660/abc.20250772)
Supplement: *Supplemental Materials [file 0066-782x-abc-123-5-e20250772-Supp01b.pdf]

APPENDIX B

Risk factors and CAD progression.

Risk factors	OR	95%CI	P-value
**Sex (male/\female)**	2.412	1.87- 2.86	<0.0001
**Age (Years)**	1.056	1.01 - 1.09	<0.0001
**Diabetes**	1.321	0.22 - 2.36	0.60
**Antidiabetes medication**	1.851	0.41 - 2.92	0.259
**Hypertension**	2.465	0.68 - 4.15	0.2840
**Anti-Hypertensives**	0.427	-1.79 – 2.08	0.312
**Dyslipidemia**	1.379	-0.29 - 2.64	0.618
**Statin**	1.428	0.47 – 2.60	0.551
**Age ≥ 65 years-old**	0.865	0.24 – 1.69	0.702

Legend: A multivariate logistic regression was performed with variables that have been qualified as risk factors since the Franingham study, and those with p<0,20 in univariate analysis (not shown). Thus, sex, age in years and dichotomized at 65 years, hypertension, diabetes mellitus, dyslipidemia and medications (antihypertensives, antidiabetics and statins) were included in the model.

Source: Prepared by the authors (2025).
