# Supplementary material for: Escore de Cálcio das Artérias Coronárias, Fatores de Risco e Desfechos Clínicos na Doença Arterial Coronariana Não Obstrutiva: Um Estudo de Seguimento de Longo Prazo
Source: Arq Bras Cardiol. 2026 Jun 12;123(5):e20250772. [Article in Portuguese] doi: 10.36660/abc.20250772 (PMC13399544; doi:10.36660/abc.20250772)
Supplement: Translation

# Translation: Coronary Artery Calcium Score, Risk Factors, and Clinical Outcomes in Nonobstructive Coronary Artery Disease: A Long-Term Follow-Up Study

**DOI:** 10.36660/abc.20250772i

**Authors:** Protásio Lemos Da Luz, Desiderio Favarato, Alexandre A.C. Abizaid, Luiz Antônio Machado Cesar, Carlos Vicente Serrano, Antônio Carlos Palandri Chagas, Carlos E. Rochitte, Marco A. Gutierrez

**Affiliations:** 1 São Paulo SP Brazil Instituto do Coração do Hospital das Clínicas da Faculdade de Medicina da Universidade de São Paulo, São Paulo, SP – Brazil

**Keywords:** Calcium, Coronary Vessels, Coronary Artery Disease

## Abstract

**Background:**

The prognostic value of coronary artery calcium (CAC) score in nonobstructive coronary artery disease (NObCAD; stenosis < 50%) remains insufficiently characterized.

**Objectives:**

To investigate the association between CAC score, cardiovascular risk factors, and clinical outcomes in patients with NObCAD.

**Methods:**

A total of 2,509 patients underwent coronary computed tomography angiography (CTA) and were followed for 8.9 ± 2.6 years. Plaque burden was classified according to CAD-RADS™ 2.0 into none, mild, moderate, and high/highest. The primary endpoint (PEP) was a composite of all-cause mortality, acute coronary syndrome/acute myocardial infarction, and stroke. Statistical significance was set at 5%.

**Results:**

CAC score was 0 in 45.4% of patients, 1-99 in 36.6%, and ≥ 100 in 18.0%. Correspondingly, 38.3% of patients had no coronary lesions, 38.5% had mild lesions, 14.1% had moderate lesions, and 9.2% had high/highest lesions. Among patients with CAC = 0, the absence of coronary lesions predominated (81.2%), whereas it was rare among those with CAC ≥ 100. The PEP occurred in 4.9% of patients, predominantly driven by all-cause mortality. Among the 396 patients with serial coronary CTA (mean interval: 6.5 ± 2.6 years), CAC progression (> 2.5 increase based on the square root method) was observed in 41.9%. Plaque burden increased in parallel. CAC score showed a positive association with plaque burden and cardiovascular risk factors.

**Conclusions:**

In NObCAD, CAC is present in more than half of patients and is associated with both plaque burden and cardiovascular risk factors. The incidence of PEP increases in proportion to risk factor burden and CAC levels. CAC and plaque burden progress concurrently over time, supporting the role of CAC as a surrogate marker of subclinical atherosclerosis progression.

## Introduction

Coronary artery calcium (CAC) is a well-established marker of atherosclerosis and a robust prognostic indicator in coronary artery disease (CAD). In addition, it improves risk stratification when incorporated into traditional cardiovascular risk models.^1–4^ Calcification is an active biological process, largely driven by ectopic bone formation, and reflects the interplay among oxidative stress, inflammation, and vascular remodeling.^5,6^ However, the absence of CAC does not exclude atherosclerosis, as many high-risk plaques remain non-calcified.^7,8^ CAC is associated with traditional risk factors, overall plaque burden, and cardiovascular events, particularly at values exceeding 300 Agatston units.^9–12^

Despite its established role, CAC has been insufficiently investigated in patients with nonobstructive CAD (NObCAD), particularly in the context of long-term follow-up. The prevalence of NObCAD varies widely and is consistently higher in women than in men.^13–15^ In patients with stable CAD, its prevalence has been reported to reach 67%.^13^ Regarding clinical outcomes, Wang et al.^13^ reported annualized event rates of 0.3% in individuals without CAD, 0.7% in those with NObCAD, and 2.7% in patients with obstructive CAD (ObCAD). Overall, the risk associated with NObCAD is approximately 72% lower than that observed in ObCAD.^13^

Historically, patients with NObCAD have been considered at low risk. However, more recent evidence challenges this assumption, which demonstrates NObCAD is associated with all-cause mortality, acute myocardial infarction (AMI), and stroke.^8,16^

Data on NObCAD and CAC in the Brazilian population remain limited or absent. This population is characterized by a unique genetic background resulting from admixture among individuals of African, European, and Indigenous American ancestry.^17,18^ Prior studies, particularly those led by Mayana Zatz, have highlighted the importance of this genetic diversity, which may influence phenotypic expression. Therefore, findings derived from other populations may not be fully generalizable to Brazilian individuals.

Accordingly, the current study assessed the presence and clinical significance of CAC score, as well as its progression over time, in a cohort of Brazilian patients with NObCAD during long-term follow-up (Central Illustration).

## Methods

### Population

The study population consisted of 2,509 consecutive patients aged 18-80 years, including both men and women, who underwent coronary computed tomography angiography (CTA) at Instituto do Coração (InCor), Faculdade de Medicina da Universidade de São Paulo, Brazil, for the evaluation of chest pain or suspected CAD, between January 2012 and December 2017. All patients were part of the InCor institutional registry.

A total of 803 patients (32.0%) were followed through in-person clinical visits at InCor. The remaining 1,706 patients (68.0%) were contacted by telephone or email and, after providing informed consent, completed a standardized questionnaire (Appendix A) regarding clinical outcomes. In cases of death, information was obtained from a relative or the patient's primary physician. As the specific causes of death were often difficult to determine, only all-cause mortality was considered.

Exclusion criteria included prior acute coronary syndrome (ACS) at baseline, prior myocardial revascularization, valvular heart disease, cardiomyopathies, chronic obstructive pulmonary disease, chronic kidney disease, malignancy, liver failure, or any condition associated with a life expectancy < 5 years.

The primary endpoint (PEP) was a composite of incident all-cause mortality, ACS/AMI, and stroke. Secondary endpoints corresponded to the individual components of the PEP.

The study protocol was approved by the human research ethics committee of InCor and registered at ClinicalTrials.gov under registry code NCT05392491.

### Coronary analysis

Coronary CTA was performed using a 320-detector row scanner (16 cm coverage; Canon Aquilion) in 90% of patients and a 64-detector row scanner in the remaining 10%, following standard acquisition protocols. CAC score was performed prior to CTA using electrocardiography-triggered axial acquisition with a slice thickness of 3 mm, without overlap or gaps.

Calcification was defined as a hyperattenuating lesion > 130 Hounsfield units with an area ≥ 1 mm^2^ or involving at least three contiguous pixels. CAC score was quantified using the Agatston method.^19^

Coronary CTA images were interpreted by experienced operators using standardized techniques. Each case was independently reviewed by two trained analysts, who evaluated reports generated by two experienced angiographers. In cases of discrepancy, the images were reassessed by two senior cardiologists to achieve consensus. Interobserver variability was 2.2%. No CTA-related complications were reported.

At the discretion of the primary physician, 396 patients (15.7%) underwent a second CTA after a mean interval of 6.5 ± 2.6 years. Indications for repeat imaging included routine follow-up, abnormal functional ischemia testing, cardiac symptoms, or preoperative evaluation for noncardiac surgery (71.6%); in 28.8% of cases, the indication was not available.

CAC progression was assessed using the square root (SQRT) method and defined as an increase > 2.5 in the SQRT-transformed CAC score between the second and first CTA. This method has been shown by Budoff et al.^20^ to be a sensitive measure of CAC progression. Plaque burden, risk factors, and clinical outcomes were subsequently compared between progressors and nonprogressors.

### Coronary territory classification

For analysis, the coronary circulation was divided into the following territories: i) left main coronary artery and left anterior descending artery, including diagonal and septal branches; ii) circumflex artery and marginal branches (Mg1 and Mg2); and iii) right coronary artery and its branches.

### Coronary artery disease classification and plaque burden

Patients were categorized into the following groups: i) no apparent CAD and ii) presence of CAD, classified according to a modified CAD-RADS™ 2.0 system.^21^

Lesion severity was defined in the following manner: i) none (0% stenosis); ii) mild (≤ 30% stenosis); and iii) moderate (> 30%-49% stenosis).

Plaque burden was classified as follows: i) none: no lesions; ii) mild: up to two mild lesions (≤ 30%); iii) moderate: three mild lesions (≤ 30%) or up to two moderate lesions (> 30%-49%); iv) high: left main involvement or at least three moderate lesions (> 30%); v) highest: progression beyond the "high" category or the presence of at least one lesion ≥ 50% on the second CTA.

Coronary lesions were visually assessed based on the luminal diameter of the diseased segment relative to the most normal-appearing proximal reference segment.

### Demographics

The following baseline data were collected at study entry (Table 1 and Appendix B): age, sex, weight, blood pressure, and height. Body mass index (BMI) was calculated from weight and height.

**Table 1:** Baseline demographic and clinical characteristics of the study population (n = 2,509)

Characteristic		n (%)	Missing
**Sex**			
	Female	1,167 (46.5%)	—
	Male	1,342 (53.5%)	—
**Race**			337
	White	1,924 (88.6%)	—
	Black	81 (3.7%)	—
	Brown	90 (4.1%)	—
	Asian	77 (3.5%)	—
**Age, median (IQR), years**		58 (50.43-64.60)	1
**Positive family history of CAD**		1,201 (56.1%)	367
**DM**		896 (35.7%)	—
**Dyslipidemia**		1,817 (72.4%)	—
**Hypertension**		1,788 (71.3%)	—
**Sedentary lifestyle**		1,105 (47.3%)	171
**Obesity**		638 (27.0%)	146
**Tobacco use**			101
	Former smoker	772 (32.1%)	—
	Current smoker	199 (8.6%)	—
**Plaque burden classification**			—
	None	961 (38.3%)	—
	Mild	963 (38.4%)	—
	Moderate	353 (14.1%)	—
	High/highest	232 (9.2%)	—
**Medications**			—
	Antihypertensive agents	1,722 (68.6%)	—
	Antidiabetic agents	837 (33.4%)	—
	Lipid-lowering therapy	1,706 (68.1%)	—

Source: Prepared by the authors (2025). CAD: coronary artery disease; DM: diabetes mellitus; IQR: interquartile range.

Hypertension was defined as systolic blood pressure > 130/85 mmHg or the use of antihypertensive medication. Obesity was defined as BMI ≥ 30 kg/m^2^ and overweight as BMI between 26 and 29 kg/m^2^. Physical activity was categorized as sedentary or active (≥ three sessions per week). A positive family history was defined as at least one parent with cardiovascular events or interventions before the age of 60 years.

Smoking status was classified as never, former, or current (≥ 10 cigarettes/day). Diabetes mellitus (DM) was defined as fasting plasma glucose ≥ 126 mg/dL, random glucose > 140 mg/dL, 2-hour glucose tolerance test > 200 mg/dL, HbA1c > 6.5%, or use of glucose-lowering medications. Dyslipidemia was defined as low-density lipoprotein cholesterol > 130 mg/dL, triglycerides > 150 mg/dL, or use of lipid-lowering therapy.

### Statistical analyses

Categorical variables are presented as absolute and relative frequencies, whereas continuous variables are expressed as mean ± standard deviation for normally distributed data or median and interquartile range for nonnormally distributed data, as assessed by the Kolmogorov-Smirnov test.

Baseline characteristics and clinical events were initially compared without adjustment. The incidence of the PEP was evaluated using Kaplan-Meier survival curves and compared with the log-rank test. Time from the initial examination to the occurrence of the primary event was used for survival analysis.

Associations between categorical variables were assessed using the chi-square test, and differences in plaque burden were evaluated using the Mann-Whitney test. Changes in CAC score between the first and second measurements, expressed as SQRT-transformed values, were analyzed using the paired Wilcoxon test, as the SQRT values did not follow a normal distribution.

Comparisons between baseline and follow-up CTA in nonprogressors and progressors were performed using the Wilcoxon signed-rank test. Binary logistic regression was used to identify factors associated with CAC progression, with results expressed as odds ratio (OR) and 95%CI. The final model was selected using a stepwise approach, including variables with p < 0.20 in univariable analysis (data not shown) or variables already established as traditional cardiovascular risk factors.

Collinearity was assessed using the variance inflation factor, with values > 4 considered indicative of collinearity. The association between CAC score and plaque burden in the context of CAC progression was evaluated using Spearman's correlation test.

## Results

### Baseline characteristics

Among the 2,509 patients, 53.5% were men and 46.5% were women, with a mean age of 57.2 ± 10.9 years. Most patients were White and had multiple cardiovascular risk factors, including hypertension, dyslipidemia, overweight/obesity, and a positive family history (Table 1).

At baseline, CAC score was 0 in 1,140 patients (45.4%), 1-99 in 917 (36.6%), and ≥ 100 in 452 (18.0%); in the latter group, the mean CAC value was 368.4 ± 347.3. Concomitantly, 961 patients (38.3%) had no coronary lesions, 963 (38.5%) had mild lesions, 355 (14.1%) had moderate lesions, and 232 (9.2%) had high/highest lesions (Figure 1).

**Figure 1:** Comparison between CAC categories and plaque burden (none, mild, moderate, high/highest) in the overall population (n = 2,509). There is a clear gradient in plaque burden across increasing CAC categories, from 0 to ≥ 100. Among patients with CAC = 0, the absence of coronary lesions predominates, whereas mild, moderate, and high/highest lesions predominate among those with CAC ≥ 100 Agatston units (Pearson's chi-square test). See text for details. CAC: coronary artery calcium.

Plaque burden at baseline was predominantly classified as none or mild, whereas only 9.2% of patients presented high/highest plaque burden (Table 1).

### Coronary artery calcium and plaque burden

There was a clear direct relationship between CAC score and plaque burden at baseline (Figure 1). Among patients with CAC = 0, the absence of coronary lesions predominated (80%), whereas no patients with CAC ≥ 100 had null lesions. Conversely, the proportions of mild, moderate, and high/highest lesions increased progressively with higher CAC values, reaching 100% among patients with CAC ≥ 100.

### Coronary artery calcium subgroups and risk factors

The number of cardiovascular risk factors increased across CAC score categories. Male sex, age > 65 years, DM, dyslipidemia, and hypertension were significantly more frequent among patients with CAC ≥ 100 compared with those with CAC = 0 (p < 0.001, chi-square test).

Although the number of risk factors increased as CAC score increased, even among patients with CAC = 0, 69.3% had three or > three risk factors (Table 2).

**Table 2:** CAC, cardiovascular risk factors, and event rates at baseline

Variable		CAC = 0 (n = 1.140)	0 < CAC < 100 (n = 917)	CAC ≥ 100 (n = 452)	Total (n)	p-value^1^
Risk factors						
**DM**		341 (29.9%)	365 (39.8%)	190 (42.0%)	2.509	< 0.001
**Dyslipidemia**		725 (63.6%)	721 (78.6%)	371 (82.1%)	2.509	< 0.001
**Hypertension**		732 (64.2%)	689 (75.1%)	367 (81.2%)	2.509	< 0.001
**Family history of CAD**		542 (54.5%)	452 (58.6%)	207 (54.9%)	2.142	0.201
**Current smoking**		93 (8.4%)	68 (7.8%)	38 (8.7%)	2.408	0.809
**Sedentary lifestyle**		496 (47.3%)	411 (47.7%)	198 (46.4%)	2.338	0.906
**Age > 65 years**		140 (12.3%)	259 (28.2%)	199 (44.0%)	2.508	< 0.001
**Male sex**		539 (47.3%)	494 (53.9%)	309 (68.4%)	2.509	< 0.001
**Risk factor burden**						< 0.0001
	0	17 (1.5%)	1 (0.1%)	0 (0.0%)	—	—
	1	101 (8.9%)	30 (3.3%)	8 (1.8%)	—	—
	2	232 (20.3%)	118 (12.9%)	32 (7.0%)	—	—
	3	327 (28.7%)	223 (24.3%)	97 (21.5%)	—	—
	> 3	463 (40.6%)	545 (59.4%)	311 (69.7%)	—	—
**Event rates by CAC category**						
	PEP	42 (3.7%)	44 (4.8%)	37 (8.2%)	—	< 0.0001
	ACS/AMI	15 (1.3%)	14 (1.5%)	10 (2.2%)	—	0.365
	Stroke	17 (1.5%)	19 (2.1%)	13 (2.9%)	—	0.141
	All-cause mortality	14 (1.2%)	12 (1.3%)	19 (4.2%)	—	< 0.0001

Participants were stratified according to CAC categories (0, 0 < CAC < 100, and CAC ≥ 100). Values are expressed as absolute numbers and percentages. Group comparisons were performed using the chi-square test1. Event-free survival was analyzed using Kaplan-Meier curves with the log-rank test. AMI: acute myocardial infarction; ACS: acute coronary syndrome; CAC: coronary artery calcium; CAD: coronary artery disease; DM: diabetes mellitus; PEP: primary endpoint.

### Correlates of coronary artery calcium progression

Among 396 patients (15.8% of the total cohort) who underwent a second CTA after a mean interval of 6.5 ± 2.6 years, 165 (41.7%) were classified as progressors according to the SQRT method, whereas 231 (58.3%) were nonprogressors.

The median (interquartile range) CAC score increased from 0 (0-31) at baseline to 12.6 (5.0-126.75) at follow-up (p < 0.0001, Wilcoxon signed-rank test).

Regarding transitions between CAC score categories, 71.9% of patients with CAC = 0 remained unchanged, 26.7% progressed to CAC score 1-99, and 1.4% progressed to CAC ≥ 100. Among patients with CAC score 1-99, 31.0% progressed to CAC ≥ 100.

Baseline characteristics of progressors and nonprogressors are shown in Table 3. In univariable analysis, male sex, age (both as a dichotomous variable > 65 years and as a continuous variable), hypertension, DM, dyslipidemia, and the use of antihypertensive, antidiabetic, and statin therapies were associated with CAC progression (Table 3). In multivariable analysis, only male sex and age remained independently associated with progression (Appendix B).

**Table 3:** Demographic and clinical characteristics of nonprogressors and progressors

Characteristic		Nonprogressors (n = 231)	Progressors (n = 165)	OR (95%CI)	p-value	Missing
**Number (%)**		231 (58.3%)	165 (41.7%)	—	—	—
**Time to second CTA, years (mean ± SD)**		6.49 ± 2.55	6.87 ± 2.66	—	0.264	0
**Sex (male/female), n (%)**		104/127 (45.0/55.0)	102/63 (61.8/38.2)	1.98 (1.32-2.97)	0.0009	0
	Age ≥ 65 years, n (%)	32 (13.9%)	38 (23.0%)	1.86 (1.11-3.13)	0.018	0
	Age, years (mean ± SD)	52.38 ± 3.68	57.79 ± 9.38	—	< 0.0001	0
**Family history of CAD, n (%)**		116 (57.1%)	80 (59.7%)	1.11 (0.71-1.73)	0.640	59
	Hypertension, n (%)	80 (34.6%)	125 (75.8%)	5.89 (3.77-9.23)	< 0.0001	0
	DM, n (%)	59 (25.5%)	77 (46.7%)	2.55 (1.67-3.90)	< 0.0001	0
	Dyslipidemia, n (%)	175 (75.8%)	144 (87.3%)	2.19 (1.27-3.80)	0.004	0
**Smoking status, n (%)**						**37**
	Current	14 (6.5%)	16 (11.1%)	1.76 (0.83-3.72)	0.136	—
	Former	52 (24.2%)	43 (29.9%)	1.29 (0.80-2.08)	0.287	—
	Never	150 (70.3%)	85 (59.0%)	0.68 (0.43-1.06)	0.085	37
**Physical activity, n (%)**		**91 (50.0%)**	**79 (56.0%)**	**1.27 (0.82-1.98)**	**0.280**	**73**
**Medications, n (%)**						
	Antihypertensive agents	148 (64.1%)	121 (73.3%)	1.54 (0.99-2.39)	0.052	0
	Antidiabetic agents	48 (20.7%)	70 (42.4%)	2.81 (1.80-4.38)	< 0.0001	0
	Statin therapy	148 (64.1%)	139 (84.2%)	2.46 (1.48-4.08)	0.0004	0

Características demográficas e clínicas comparando não progressões (n = 231) e progressões (n = 165). Algumas variáveis apDemographic and clinical characteristics comparing nonprogressors (n = 231) and progressors (n = 165). Some variables had missing data, including family history, smoking status, and physical activity. Time to second CTA was normally distributed (Kolmogorov-Smirnov test). Source: Prepared by the authors (2025). CAD: coronary artery disease; CTA: computed tomography angiography; DM: diabetes mellitus; OR: odds ratio; SD: standard deviation.

CAC score increased significantly from the first to the second CTA among progressors (from 120.4 ± 206.3 to 283.4 ± 396.7 Agatston units; p < 0.001). A smaller but statistically significant increase was also observed among nonprogressors (from 27.0 ± 120.0 to 32.3 ± 131.6 Agatston units; p < 0.0001) (Figure 2A-B).

**Figure 2:** A) Baseline and follow-up CAC in progressors; B) baseline and follow-up CAC in nonprogressors; C) baseline and follow-up plaque burden in progressors (n = 165); D) baseline and follow-up plaque burden in nonprogressors (n = 231). CAC progression was defined as an increase ≥ 2.5 in the SQRT-transformed CAC between the first and second CTA. Both groups exhibited significant changes in plaque burden at follow-up compared with baseline, although the magnitude of change was smaller in nonprogressors (McNemar test). CAC: coronary artery calcium; CTA: computed tomography angiography; SQRT: square root.

Using SQRT transformation, progressors exhibited an increase from 5.1 (0.0-12.47) to 11.45 (6.02-18.99) (p < 0.0001), exceeding the predefined cutoff of 2.5. In contrast, nonprogressors showed a smaller increase, from 0.0 (0.0-0.0) to 0.0 (0.0-2.16) (p < 0.001), remaining below the cutoff (Figure 2A-B).

Final CAC score and plaque burden values were significantly higher in progressors than in nonprogressors (p < 0.001 for both; chi-square and Mann-Whitney tests) (Figure 3). Overall, both CAC score and plaque burden increased over time, with greater increases observed among progressors.

**Figure 3:** Comparison of follow-up CAC and plaque burden between progressors and nonprogressors. Progressors exhibited significantly higher CAC values and greater plaque burden compared with nonprogressors (Mann-Whitney test; p < 0.001 for both). See text for details. CAC: coronary artery calcium; SQRT: square root.

A positive correlation was observed between changes in CAC score and plaque burden in both progressors and nonprogressors (r = 0.571 and r = 0.558, respectively; p < 0.001 for both, Spearman test).

### Coronary artery calcium and primary endpoint

Overall, 123 patients (4.9%) experienced the PEP over a mean follow-up of 8.9 ± 2.6 years. The distribution of events (all-cause mortality, ACS/AMI, and stroke) is presented in Table 2.

In multivariable analysis including CAC categories and all risk factors, family history, age > 65 years, and sedentary lifestyle were independently associated with PEP (Table 4). When CAC score categories and number of risk factors were analyzed separately, both were significantly associated with events (CAC: OR, 1.36, 95%CI, 1.2-1.6; p = 0.012; risk factors: OR, 1.56, 95%CI, 1.3-1.8; p = 0.0007) (Table 4).

**Table 4:** Multivariable logistic regression analysis for the PEP

Variable	OR	95%CI	p-value
Model 1			
CAC categories	1.11	0.80-1.53	0.512
Male sex	1.66	1.21-2.27	0.027
Family history of CAD	2.20	1.73-3.00	0.001
DM	1.17	0.73-2.00	0.481
Hypertension	1.70	1.13-2.57	0.070
Dyslipidemia	0.73	0.24-1.20	0.212
Current smoking	1.70	1.00-2.90	0.140
Sedentary lifestyle	2.33	1.89-3.00	< 0.0001
Age ≥ 65 years	4.72	4.24-5.26	< 0.0001

OR represent the increase in risk per category increment. CAC categories were defined as 0, 1-99, and ≥ 100. Risk factor burden was categorized as 0, 1, 2, 3, and > 3 factors. Source: Prepared by the authors (2025). CAC: coronary artery calcium; CAD: coronary artery disease; DM: diabetes mellitus; OR: odds ratio; PEP: primary endpoint.

Among the 165 progressors, four events (2.4%) occurred, compared with six events (2.6%) among the 231 nonprogressors, with no significant difference between groups.

Event rates were similar between men (7.2%) and women (6.1%) (p = 0.26). However, patients aged > 65 years (n = 598) had a higher incidence of events compared with younger individuals (12.4% vs 4.9%; p < 0.001).

## Discussion

In the present study, CAC was a frequent finding among patients with NObCAD, with approximately half of the cohort presenting CAC > 0 and 18.0% exhibiting CAC ≥ 100. CAC score was directly associated with plaque burden, traditional cardiovascular risk factors, and disease progression. Notably, this study was conducted in a Brazilian population characterized by a unique genetic background resulting from admixture among individuals of African, European, and Indigenous ancestry.^17,18^ This feature adds originality to our findings, as comparable long-term studies in similar populations remain scarce. Prior large-scale studies with extended follow-up have demonstrated that increased CAC values are associated with major cardiovascular events, including all-cause mortality.^7,8^

Conversely, prior investigations have emphasized the prognostic value of CAC = 0, both as a negative predictor of cardiovascular events^22,23^ and as a modifier of therapeutic decisions, including the use of statins and semaglutide.^24,25^ However, our findings differ slightly, as the incidence of PEP among patients with CAC = 0 was not negligible (3.7%). This observation is likely explained by the high prevalence of risk factors in this subgroup, particularly when ≥ three risk factors were present, as well as the relatively long follow-up period (8.9 years). These findings reinforce that CAC score should not be interpreted in isolation but rather in conjunction with the overall risk factor profile. Importantly, the absence of CAC does not exclude the presence of atherosclerotic plaques, including potentially high-risk lesions.

The consistent, robust relationship observed between CAC and plaque burden in this study confirms previous reports.^1–3^ While CAC = 0 was predominantly associated with absent or mild plaque burden, CAC ≥ 100 was strongly associated with moderate to high plaque burden. Rumberger et al.^11^ demonstrated a direct and statistically significant relationship between CAC score and plaque burden using SQRT transformation to account for skewed data distribution. In addition, CAC was associated with older age, DM, hypertension, and dyslipidemia, in agreement with prior studies.^10,12^

Progression of CAC was observed in 41.7% of patients who underwent a second CTA and was associated with advanced age, dyslipidemia, DM, and hypertension, but not with other risk factors. Progression was defined using the SQRT method, which is considered a sensitive indicator of atherosclerosis progression.^19^

Similarly, Fuster et al.,^26^ in a cohort of 732 asymptomatic individuals followed for 12.4 years, demonstrated that both carotid plaque burden and CAC were associated with all-cause mortality and progression of atherosclerosis. These findings are consistent with those of Eghtedari et al.,^27^ who studied 3,260 patients with a mean interval of 4.7 ± 3.2 years between scans and a follow-up of 9 years, showing that an annual increase of 20 Agatston units predicted all-cause mortality.

The potential influence of pharmacological therapies, including antihypertensive, antidiabetic, and lipid-lowering agents, cannot be excluded. In univariable analysis, such associations were observed. Specifically, Rosendael et al.^28^ reported that statin therapy may attenuate plaque progression. However, in multivariable analysis, only male sex and age remained independently associated with CAC progression. Because of such uncertainties, likely related to the limited sample size of patients with serial imaging, definitive conclusions cannot be drawn.

Notably, changes in CAC score were positively correlated with changes in plaque burden in both progressors and nonprogressors, with a stronger association among progressors (Table 3). Although CAC progression may serve as a surrogate marker of plaque evolution, this was not reflected in clinical outcomes, as similar PEP rates were observed in both groups. These findings suggest that increases in CAC reflect subclinical atherosclerosis progression. Moreover, the association between CAC progression and risk factors underscores the importance of optimal risk factor control to prevent disease progression.

Overall, our findings are consistent with previous studies demonstrating NObCAD is associated with adverse cardiovascular outcomes, including death and AMI.^14,15^ CAC was significantly associated with clinical events, with higher event rates observed among patients with CAC ≥ 100 compared with those with CAC = 0. Specifically, 3.7% of patients with CAC = 0 experienced events, compared with 8.2% among those with CAC > 0. These findings are consistent with the MESA study^3^ and align with observations in patients with ObCAD.

The parallel progression of CAC score and plaque burden suggests routine repetition of CTA shortly after the initial examination may not be necessary, except in selected clinical scenarios.

### Strengths and limitations of study

This study includes a large cohort (n = 2,509) with long-term follow-up (mean 8.9 years), encompassing both men and women across a wide age range and without prior cardiovascular events. The observed association between CAC score and clinical outcomes reinforces its role as a marker of atherosclerosis and highlights its potential utility in guiding preventive strategies, particularly in individuals who may otherwise be considered at low risk based on clinical assessment alone. In addition, this study provides novel data on CAC as a marker of atherosclerosis progression in patients with NObCAD within a Brazilian population.

However, several limitations should be acknowledged. Data collection relied partly on self-reported information, which may introduce reporting bias. The study was conducted at a single center, limiting generalizability to other populations. Furthermore, the number of patients undergoing repeat CTA was relatively small, precluding definitive conclusions regarding the relationship between disease progression and clinical events. It remains unclear whether clinical events were driven by plaque progression or by plaque instability related to microvascular endothelial dysfunction and/or thrombosis.

Finally, this was a pragmatic, nonrandomized study in which the indication for CTA was based on physician discretion. Only patients with NObCAD were included and followed over a long-term period. Within this context, the present findings may contribute to more individualized management strategies for patients with subclinical CAD.,

## Conclusions

CAC is a marker of coronary atherosclerosis and its clinical complications, as well as a robust prognostic indicator. It is closely associated with plaque burden and traditional cardiovascular risk factors.

In addition, CAC score and plaque burden exhibit parallel behavior in both the progression and stabilization of CAD. Importantly, CAC score should not be interpreted in isolation but rather in conjunction with the overall risk factor profile.

These findings provide further evidence on the role of CAC score in NObCAD and may have implications for clinical decision-making.

## Data Availability

All datasets supporting the results of this study are available upon request from the corresponding author

## *Supplemental Materials

*Supplemental Materials For Appendix A, please click here. For Appendix B, please click here.
